# Magnetic resonance imaging of meningiomas: a pictorial review

**DOI:** 10.1007/s13244-013-0302-4

**Published:** 2014-01-08

**Authors:** J. Watts, G. Box, A. Galvin, P. Brotchie, N. Trost, T. Sutherland

**Affiliations:** St Vincent’s Hospital, 41 Victoria Pde, Fitzroy, Victoria 3065 Australia

**Keywords:** Meningioma, Diagnosis, Magnetic resonance imaging, Spectroscopy, Diffusion tensor imaging

## Abstract

Meningiomas are the most common non-glial tumour of the central nervous system (CNS). There are a number of characteristic imaging features of meningiomas on magnetic resonance imaging (MRI) that allow an accurate diagnosis, however there are a number of atypical features that may be diagnostically challenging. Furthermore, a number of other neoplastic and non-neoplastic conditions may mimic meningiomas. This pictorial review discusses the typical and atypical MRI features of meningiomas and their mimics.

- *There are several characteristic features of meningiomas on MRI that allow an accurate diagnosis*
- *Some meningiomas may display atypical imaging characteristics that may be diagnostically challenging*
- *Routine MRI sequences do not reliably distinguish between benign and malignant meningiomas*
- *Spectroscopy and diffusion tensor imaging may be useful in the diagnosis of malignant meningiomas*
- *A number of conditions may mimic meningiomas; however, they may have additional differentiating features*

## Background

Meningiomas are the most common non-glial tumours of the central nervous system (CNS), accounting for between 16 and 20 % of all intracranial tumours [1]. Magnetic resonance imaging (MRI) is the modality of choice for the investigation of meningiomas, providing superior contrast differentiation and usually the ability to differentiate between intra- and extra-axial lesions. In addition to MRI, computed tomography (CT) has a useful role in specific cases where there is calcification and adjacent changes to the calvarium. Although typical meningiomas have characteristic imaging features, there are multiple atypical variants that may be diagnostically challenging and the value of MRI in predicting WHO grades in meningiomas is limited. A number of benign and malignant pathologies may also mimic some of the features of meningiomas. It is important for the radiologist to have an understanding of the typical and atypical features of meningiomas to aid their recognition and suggestion of a correct diagnosis. The typical and atypical imaging appearance of meningiomas is discussed.

## Epidemiology

Meningiomas have an annual incidence of 6 per 100,000 and are twice as common in the female as in the male population [2]. They are most common after the 5th decade of life and are often asymptomatic, with a 2–3 % prevalence in the elderly population. When symptomatic, meningiomas present with a wide variety of symptoms, arising from compression of adjacent structures, direct invasion of or reactive changes in the brain or due to obstruction of cerebrospinal fluid (CSF) pathways or vessels.

The majority of meningiomas are spontaneous and of unknown aetiology, although recognised risk factors include previous exposure to radiation, genetic disorders such as neurofibromatosis type 2, in which the tumours may be multiple [3], and after head injury, although the causality in the latter is unclear [2].

## Neuropathological features

Meningiomas are typically slow-growing tumours that arise from the meningothelial cells of the arachnoid. Histological grading of meningiomas is based on the current WHO classification. The majority of lesions are benign WHO Grade I lesions, representing approximately 90 % of cases. The histological subtypes of grade I meningiomas include meningothelial, psammomatous, secretory, fibroblastic, angiomatous, lymphoplasmacyte-rich, transitional, metaplastic and microcystic. They differ from the more aggressive meningiomas, WHO grade II (atypical) and WHO grade III (anaplastic), 5–7 % and 1–3 % of cases respectively [2], in their number of mitoses, cellularity, nuclear-to-cytoplasmic ratio, histological patterns and their relatively low risk of recurrence or aggressive growth pattern [4].

## Lesions and locations

**Fig. 1:**
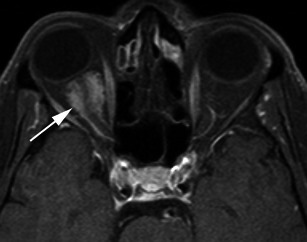
Post-contrast axial T1-weighted sequence in a 64-year-old man with proptosis demonstrates a homogeneously enhancing, intraconal mass within the right orbit (*arrow*) that partially encircles the right optic nerve. Smooth dural thickening and enhancement extends posteriorly to the optic canal. The imaging appearance is consistent with a meningioma

**Fig. 2:**
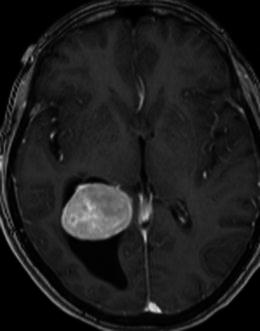
Post-contrast axial T1-weighted sequence in a 62-year-old woman with headaches demonstrates a large, homogeneously enhancing mass within the trigone of the right lateral ventricle associated with ventricular dilatation. Histology was that of a meningioma (grade I)

**Fig. 3:**
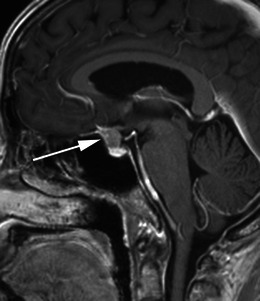
Post-contrast sagittal T1-weighted sequence in a 52-year-old woman with reduced vision in the left eye demonstrates a homogeneously enhancing suprasellar mass (*arrow*) extending inferiorly into the superior aspect of the sella where it contacts the brightly enhancing anterior pituitary. The mass results in mild elevation of the left side of the optic chiasm

**Fig. 4:**
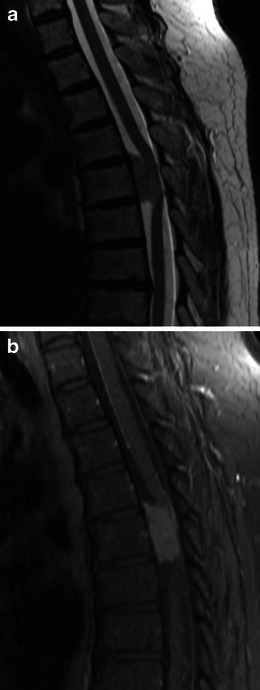
**a** Sagittal T2-weighted sequence in a 68-year-old woman with mild weakness and dysaesthesia of the left leg demonstrates an intradural, extramedullary mass anteriorly at T5, which displaces the spinal cord posteriorly, resulting in severe narrowing of the central spinal canal. **b** Post-contrast sagittal T1-weighted sequence in the same patient as **a** demonstrates homogeneous enhancement. The histological diagnosis at surgery was a meningioma

Meningiomas may be found along any of the external surfaces of the brain as well as within the ventricular system where they arise from the stromal arachnoid cells of the choroid plexus. The most common locations include the parasagittal aspect of the cerebral convexity, the lateral hemisphere convexity, the sphenoid wing [5], middle cranial fossa and the olfactory groove. Meningiomas at the skull base may extend through foramina, for example into the orbit and along the course of the trigeminal nerve. Meningiomas represent the second most common mass lesion of the cerebellopontine angle, secondary to the acoustic schwannoma [5]. Less common locations include the optic nerve sheath (0.4–1.3 % of cases) [3] (Fig. 1), the choroid plexus (0.5–3 % of cases) [3], most commonly in the trigone of the lateral ventricle in adults (Fig. 2), and the sella turcica (Fig. 3). Approximately 10 % of meningiomas arise in the spine (Fig. 4). Very rarely, representing approximately 1 % of cases, they may arise entirely outside the dura and may be purely extracalvarial, calvarial or calvarial with extracalvarial extension [6], with sites including the temporal bone, mandible, mediastinum and lung described [2]. The aetiology of these ectopic locations is thought to be due to meningocytes or arachnoid cap cells trapped in the cranial sutures during remoulding of the brain at birth, as a result of trauma or as a result of meningothelial differentiation from multipotential mesenchymal cell precursors [5, 6].

Meningiomas are usually solitary. In the setting of multiple meningiomas, a diagnosis of neurofibromatosis type 2 should be considered.

## Imaging characteristics

**Fig. 5:**
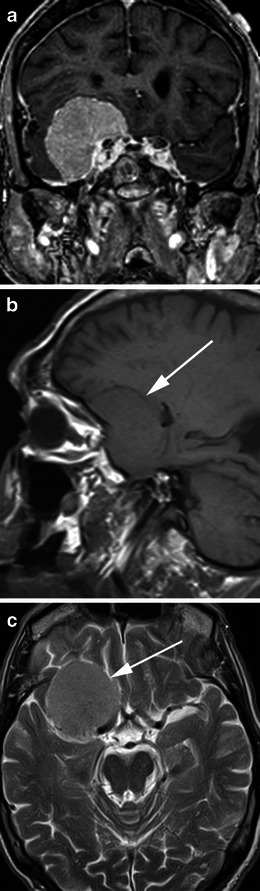
**a** Post-contrast coronal T1-weighted sequence in an 80-year-old woman with a 1-month history of confusion demonstrates a well-circumscribed, homogenously enhancing mass within the floor right middle cranial fossa with a broad dural tail that inferomedially contacts the right cavernous sinus. The right temporal lobe is compressed superiorly by the mass without invasion. The lesion was proven at surgery to be a meningioma. **b** Sagittal T1-weighted sequence in the same patient as **a** demonstrates the mass (*arrow*) to be isointense to grey matter and to extend anteriorly along the posterior aspect of the anterior cranial fossa floor. **c** Axial T2-weighted sequence in the same patient as **a** demonstrates the mass (*arrow*) to be mildly, homogeneously hyperintense to grey matter

**Fig. 6:**
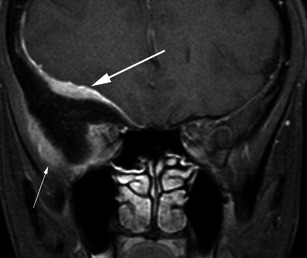
Post-contrast coronal T1-weighted sequence in a 33-year-old woman who presents with right proptosis demonstrates extensive dural thickening and enhancement along the greater wing of sphenoid consistent with an en plaque meningioma (*large arrow*). The lesion results in marked hyperostosis of the greater wing of sphenoid with resultant indentation of the right temporal lobe. Further enhancing tumour is identified external to the calvarium between the greater wing of sphenoid and the temporalis muscle (*small arrow*)

**Fig. 7:**
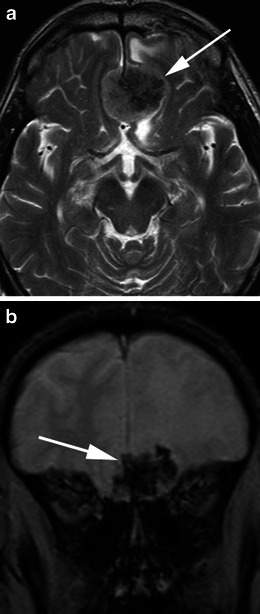
**a** Axial T2-weighted sequence in a 75-year-old man being investigated for Bell palsy demonstrates a heterogeneous, extra axial mass in the midline of the olfactory groove, left more than right, in keeping with a meningioma (*arrow*) associated with mild oedema within the left frontal lobe. There are intrinsic areas of low signal intensity in keeping with calcification that do not enhance on post contrast sequences. **b** Coronal T2* sequence in the same patient as **a** demonstrates susceptibility effect (*arrow*) within the mass in keeping with the presence of calcification

Meningiomas typically appear as lobular, extra axial masses with well-circumscribed margins (Fig. 5a). They typically have a broad-based dural attachment and, if sufficiently large, inward displacement of the cortical grey matter [5]. They may occasionally, however, exhibit a more infiltrating growth pattern over the dura, termed meningioma en plaque (Fig. 6), which most commonly occur along the sphenoid ridge or the convexity. The typical MRI signal intensity characteristics consist of isointensity to slight hypointensity relative to grey matter on the T1-weighted sequence (Fig. 5b) and isointensity to slight hyperintensity relative to grey matter on the T2 sequence (Fig. 5c). After contrast administration, meningiomas typically demonstrate avid, homogeneous enhancement (Fig. 5a); however, they may occasionally have areas of central necrosis or calcification that do not enhance. Calcification is typically best demonstrated on CT, with variable reported rates of occurrence. On MRI, calcification is best identified on susceptibility weighted images as areas of low signal intensity (Fig. 7b); however calcification may also be appreciated on T2-weighted sequences as areas of low signal intensity (Fig. 7a). Contrast is especially useful in delineating en plaque meningiomas [2] that are typically seen as asymmetric thickened sheets of enhancing dura (Fig. 6).

**Fig. 8:**
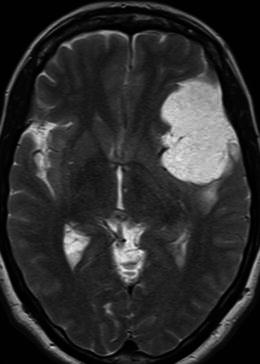
Axial T2-weighted sequence in a 55-year-old woman with an 8-year history of syncopal attacks demonstrates a large, extra-axial mass over the inferolateral left frontal lobe and anterior temporal lobe that is markedly hyperintense, slightly more so than CSF. There is mild vasogenic oedema present posterior to the mass. Histology was consistent with a microcystic meningioma

The rare microcystic subtype of meningioma, representing 1.6 % of intracranial meningiomas, is different in its signal characteristics and typically demonstrates uniform low signal intensity on T1-weighted and high signal intensity on T2-weighted sequences (Fig. 8). In a recent study reviewing the radiological appearance of 16 histologically proven cases, Paek et al. [7] described dense, homogeneous enhancement in 11, heterogeneous enhancement in 3 and 2 cases of scant, peripheral enhancement.

**Fig. 9:**
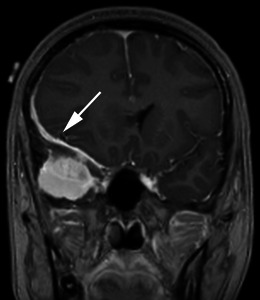
Post-contrast coronal T1-weighted sequence in a 35-year-old woman with headaches demonstrates a homogenously enhancing extra axial mass within the right middle cranial fossa with a broad dural tail (*arrow*) extending along the sphenoid wing and around the lateral aspect of the right frontal lobe. The mass results in subfalcine herniation to the left by 7 mm and effacement of the right lateral ventricle. Histology at surgery was a meningioma (grade 1)

**Fig. 10:**
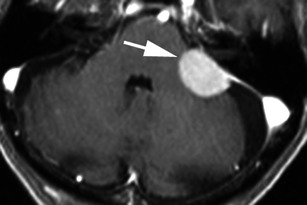
Post-contrast axial T1-weighted sequence in an 83-year-old man with dizziness and falls demonstrates a homogeneously enhancing, extra-axial mass within the left cerebellopontine angle (*arrow*) with a broad dural attachment and a dural tail extending anterior to the left internal acoustic meatus and posteriorly to the sigmoid sinus. The mass overlies the left internal acoustic meatus without intracanalicular extension. Appearance and histology are consistent with a meningioma (grade I)

**Fig. 11:**
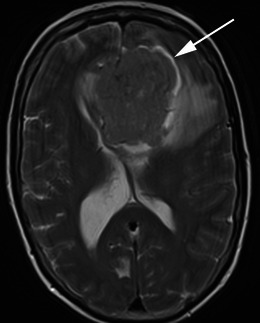
Axial T2-weighted sequence in a 57-year-old woman with confusion demonstrates a large extra-axial mass centred on the falx that is isointense to grey matter with a prominent surrounding CSF signal intensity cleft between the mass and the adjacent frontal cortex, best appreciated around the left aspect (*arrow*). The diagnosis was meningioma (grade I) at histology

Imaging with MRI may allow the identification of the dural tail, defined as enhancement of the dura infiltrating away from the lesion (Fig. 9) and described in up to 72 % of cases in one study [3]. In certain circumstances, the presence of a dural tail may be useful to distinguish meningiomas from other potential aetiologies; for example, in distinguishing meningioma from schwannoma in the cerebellopontine angle as the latter is not typically associated with a dural tail (Fig. 10). Although common with meningiomas, the dural tail sign is not specific as it is also described in some metastases, glial tumours [8] and lymphoma [3]. Another important imaging feature suggestive of an extra axial location is the presence of a CSF cleft between the tumour and the underlying brain cortex (Fig. 11). The cleft may contain CSF or cortical vessels entrapped between the tumour and the underlying cortex.

**Fig. 12:**
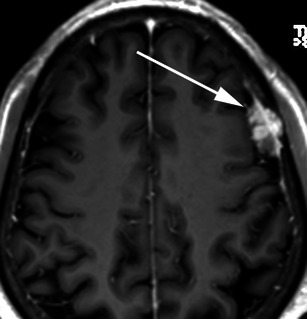
Post-contrast axial T1-weighted MRI in a 73-year-old man with headaches demonstrates a mildly heterogeneously enhancing extra-axial mass over the left lateral convexity (*arrow*) consistent with a meningioma at histology that invades the inner table and diploic space of the left frontal bone. The outer table is contacted and thinned without breach

**Fig. 13:**
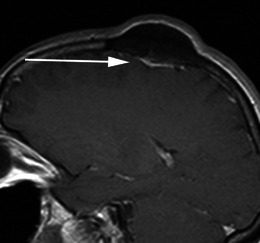
Post-contrast sagittal T1-weighted sequence in a 49-year-old woman with a long-standing palpable lump demonstrates a pronounced hyperostotic focus within the left parietal bone with the bulk protruding extracranially. Immediately below the bone lesion there is a thin sheet of homogenously enhancing extra-axial soft tissue (*arrow*) consistent with an en plaque meningioma that was proved at surgery

Bone changes associated with meningiomas include osteolysis (Fig. 12) and hyperostosis (Fig. 13), with the latter most common, described in 20 % of cases [3] and more common with the en plaque form. There may also be enlargement of the skull base foramina. The bone changes associated with meningiomas are best depicted and assessed on CT; however, they may be appreciated on MRI. Hyperostosis is most common in tumours arising from the skull base and anterior cranial fossa and the degree of hyperostosis is not proportional to tumour size. The pathological causes described include direct tumour invasion of bone and reactive hypervascularity of the periosteum leading to benign bone formation. It may difficult to distinguish radiologically between the hyperostosis associated with an en plaque meningioma and primary intraosseous meningioma that is osteoblastic (reported in 59 % of cases) [6], particularly given that the latter may be associated with underlying dural enhancement [9]. Homogeneous, dense enhancement of the tumour within the skull may help distinguish a primary intraosseous meningioma from a meningioma en plaque [9]. Histological assessment may be required to distinguish between the two conditions in other cases.

**Fig. 14:**
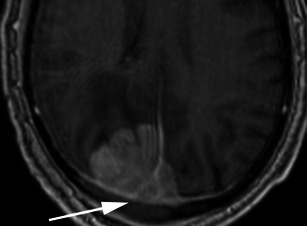
Post-contrast axial T1-weighted sequence in a 79-year-old man demonstrates a large, homogeneously enhancing extra axial mass within the right occipital lobe that extends into and expands the posterior aspect of the superior sagittal sinus (*arrow*) and crosses the midline into the paramedian left occipital lobe. Posteriorly, the mass extends into the adjacent skull with erosion of the outer table. Histology at surgery was a meningioma (grade III)

Meningiomas located at the skull base, by virtue of their position, have the potential to contact and encase vessels, particularly the carotid arteries. Whilst the encasement may be substantial and result in luminal narrowing, the reported rate of cerebrovascular insufficiency is exceedingly rare with few cases documented [10, 11]. In comparison, dural venous sinuses are much more commonly associated with tumoural invasion that may result in partial or complete sinus occlusion (Fig. 14). This is particularly the case with the superior sagittal sinus.

Magnetic resonance spectroscopy (MRS) is considered to be useful for the diagnosis of meningiomas whose radiological appearance is atypical and it may also play a role in the evaluation of malignant potential [12]. Meningiomas demonstrate increased choline (3.2 ppm) and decreased creatine (3.0 ppm). Alanine has been suggested by various studies to be specific for meningiomas; however, different studies have reported variable occurrence rates. A recent study by Yue et al. [12] demonstrated that the variance in reportable alanine levels might be due to the fact that alanine and lactate have very similar resonance peaks (1.47 ppm versus 1.33 ppm) and may partly overlap, particularly at low magnetic field intensity. Similarly, the alanine level is affected by voxel sizes, with large voxel sizes yielding stronger metabolite signals and signal-to-noise ratio. In cases of small, heterogeneous meningiomas, smaller voxel sizes may thus result in undetectable levels of alanine. In cases where alanine levels are absent or ambiguous, glutamine/glutamate (Glx, 3.75 ppm), although not a unique metabolite for meningioma, has been recognised as a potential supplementary metabolite to help detect meningiomas [12]. Lactate has also been shown in some studies to be more frequently observed in non-benign meningiomas, WHO Grade II and III, but is not always a marker of an aggressive meningioma. Similarly, lipid (Lip, 0.9/1.3 ppm), whilst usually regarded as a marker of an aggressive meningioma, does not always represent micronecrosis and therefore cannot always be taken as proof of a non-benign meningioma [12], with lipid observed in microcystic and in fatty degeneration in lipomatous tumours.

## Uncommon/rare features

A number of atypical imaging characteristics of meningiomas have been described.

**Fig. 15:**
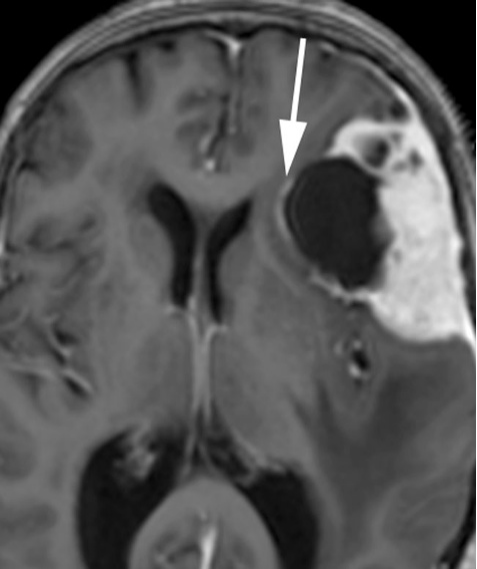
Post-contrast axial T1-weighted sequence in an 80-year-old woman with confusion demonstrates a solid and cystic, extra-axial mass overlying the left frontal lobe with a fine rim of enhancement (*arrow*) around the largest cystic component consistent with a peritumoural cyst. The homogeneously enhancing solid component demonstrated the typical isointensity to grey matter on the T1- and T2-weighted sequences of a meningioma

Cystic meningiomas are rare (Fig. 15), accounting for between 2 and 4 % of intracranial meningiomas [13]. Various classification schemes have been proposed that typically divide them into intratumoural and extratumoural in location, with one scheme by Nauta et al. describing four subtypes: intratumoural cysts completely surrounded by tumour (I), intratumoural cysts at the periphery of the tumour surrounded by a histologically detectable row of neoplastic cells (II), peritumoural cysts whose walls partly consist of tumour (III) and peritumoural cysts whose walls are formed by arachnoid and are separated from tumour by a capsule (IV). A fifth peritumoural subtype was added by Worthington et al. and comprises entrapped CSF (V) [13]. The pathogenesis of these cysts has been discussed in various studies with intratumoural cysts thought to be due to cystic degeneration, ischemic necrosis or haemorrhage [13] and peritumoural cysts thought to originate from compression of peritumoural subarachnoid space and progressive encasement of CSF spaces or due to peripheral degeneration [5, 8]. Although the differentiation between intratumoural and extratumoural cysts may be suggested by ring enhancement surrounding a tumoural cyst, histological confirmation may be required. The radiological distinction nevertheless, may aid the neurosurgeon in the decision of resectability.

**Fig. 16:**
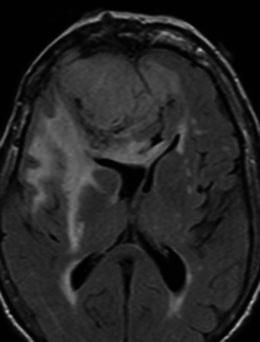
Axial FLAIR sequence in an 81-year-old woman with confusion and falls demonstrates a large extra-axial mass that is isointense to grey matter and is predominantly located within the right frontal lobe but crosses the midline falx anteriorly to indent the left frontal lobe. It induces substantial vasogenic oedema within the right frontal lobe, around the right basal ganglia and through the genu of the corpus callosum to the left forceps minor

Despite their extra-axial location, meningiomas are not uncommonly associated with brain oedema (Fig. 16), reported to be present in 60 % of all cases in one study [14]. There are several pathogenic theories regarding the development of oedema, which is currently accepted to be vasogenic rather than cytotoxic. These include: a hypothesis that tumour cells secrete an oedema-inducing substance into the adjacent brain parenchyma; a hydrodynamic theory suggesting that a pressure gradient between the extracellular space of the tumour and the interstitium of the brain results in osmotic dispersion; a theory that chronic pressure by a tumour on the brain results in ischaemia and secondary to venous obstruction by the tumour [14]. Furthermore, the development of pial and cerebral arterial perfusion of tumour, rather than purely dural arterial supply that may provide sufficient nutrition to small meningiomas, closely correlates with the incidence of oedema. The expression of vascular endothelial growth factor (VEGF) has also been suggested by Bitzer et al. [14] to be an important factor in meningioma-related vasogenic oedema, with a correlation between VEGF expression and pial blood supply.

Regardless of the mechanism of oedema production, its extent has little correlation with tumour size. There are some MRI features of meningiomas, however, that do correlate with the presence of brain oedema. The absence of a CSF cleft between tumour and brain cortex and an irregular tumour margin are both significant predictors of brain oedema and are regarded in one study to be the result of cortical penetration by the tumour [15]. Whilst these two factors are closely related to tumour size, and hence suggest size is indirectly related to the presence of oedema, these factors are themselves not enough to account for oedema formation [15]. A study by Nakano et al. [15] found that hyperintense tumours on T2-weighted MRI were accompanied by brain oedema more frequently than hypointense tumours and the oedema tended to be much more severe. This has been suggested to be due to the elevated water content in hyperintense tumours allowing more diffusion of water into the surrounding brain according to the pressure gradient theory [15]. A characteristic MRI feature of the microcystic meningioma is the high incidence of peritumoural oedema, reported in 87.5 % of cases in the study by Paek et al. [7], with the majority of those cases demonstrating oedema to a severe degree. In the study by Nakano et al. [15], no correlation was found between tumour location and brain oedema, except that brain oedema is rarely visualised in cases of posterior fossa meningiomas.

**Fig. 17:**
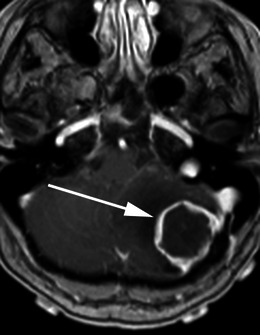
Post-contrast axial T1-weighted sequence in a 68-year-old woman with headache and ataxia demonstrates a ring enhancing mass within the left posterior cranial fossa (*arrow*) with a broad dural base posterolaterally. There is surrounding vasogenic oedema with midline shift and effacement of the fourth ventricle. Histology at surgery was a meningioma with central necrosis secondary to infarction

Meningiomas may uncommonly demonstrate an abnormal enhancement pattern post contrast administration. The enhancement may be heterogeneous secondary to the presence of intrinsic calcification, cysts and necrosis [3]. Ring enhancement may be seen in cases with central cyst formation, haemorrhage or necrosis [5] with the peripheral enhancement representing typical enhancement of the viable meningeal neoplasm (Fig. 17). A rare lipomatous or lipoblastic meningioma represents a subtype of meningioma in which there is a metaplastic change of meningothelial cells into adipocytes, resulting in a shortening of the T1 relaxation time and high signal intensity on the T1-weighted sequence [3, 5]. This subtype of meningioma often demonstrates a heterogeneous enhancement pattern [3].

**Fig. 18:**
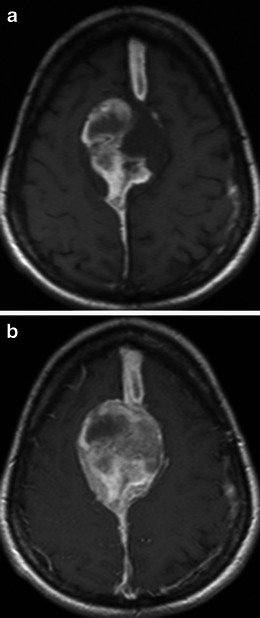
**a** Axial T1-weighted sequence in a 45-year-old woman with a 5-day history of headaches demonstrates a large, extra-axial mass centred on the falx that bulges laterally with convex margins to indent the paramedian frontal lobes bilaterally. There is heterogeneous hyperintensity within the mass consistent with intratumoural haemorrhage with subdural haemorrhage extending anteriorly and posteriorly away from the mass along the falx. Histology at surgery was a meningioma (grade I). **b** Post-contrast axial T1-weighted sequence in the same patient as **a** demonstrates mildly heterogeneous enhancement of the non-haemorrhagic component of the mass

Haemorrhage is a very rare and unusual presentation of meningiomas with very few reported cases in the literature. A study by Bosnjak et al. [16] that reviewed the clinicopathological features of 145 cases (143 literature-derived) of unsuspected meningiomas associated with spontaneous haemorrhage found there was an increased bleeding tendency with two age groups, under 30 and over 70 years, convexity and intraventricular locations and the fibrous histological subtype [16]. There was no statistically confirmed increased association with haemorrhage in the angioblastic and malignant histological subtypes. The patterns of haemorrhage include subarachnoid, subdural, intracerebral and intratumoural (Fig. 18a and b). The proposed mechanism of spontaneous haemorrhage in meningiomas are vast and include weakening of feeding and draining vessels, intratumoural angiomatous areas with friable vascular walls, meningeal invasion of vessel walls, blood dyscrasias, concurrent anticoagulation and head trauma [16].

The distinction between benign and atypical or malignant meningiomas is not reliably accomplished when assessing the imaging features on routine MRI [17]. Diffusion tensor imaging (DTI) may aid in the distinction with several studies reporting a decreased apparent diffusion coefficient (ADC) in high-grade tumours [1, 4, 17]. Various theories have been proposed to explain the reduced ADC and include a decreased free diffusion of extracellular water and the high nuclear-to-cytoplasmic ratio of high-grade tumours, resulting in a reduction in the free translation of intracellular water [4, 17]. Because atypical and malignant meningiomas are more prone to recurrence and an aggressive growth pattern, DTI may provide useful diagnostic information for surgical planning and prognostication.

**Fig. 19:**
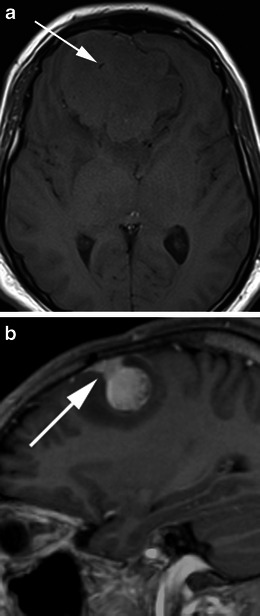
**a** Axial T1-weighted sequence in a 48-year-old man with presumed meningioma on CT demonstrates a large, extra-axial mass in the anterior cranial fossa within the interhemispheric fissure resulting in marked indentation of the medial aspect of the frontal lobes. The mass is predominantly isointense to grey matter and a number of flow voids are visualized around the periphery of the mass and centrally (*arrow*) in keeping with prominent vascularity. Histology at surgery was a haemangiopericytoma. **b** Post-contrast sagittal T1-weighted sequence in a 32-year-old man with a history of seizure post fall demonstrates an avidly enhancing, mildly heterogeneous, extra-axial mass indenting the right frontoparietal junction superiorly with a prominent pedicle (*arrow*) attached to the dura. A peripheral low signal intensity rim represents surrounding cortical grey matter and mild vasogenic oedema. Histology at surgery was a haemangiopericytoma

**Fig. 20:**
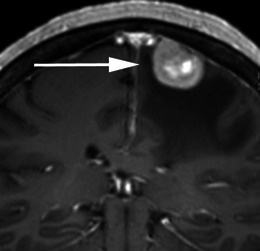
Post-contrast coronal T1-weighted sequence in a 70-year-old woman with headaches demonstrates a mildly heterogeneously enhancing mass indenting the right precentral gyrus (*arrow*) with a broad dural attachment and marked surrounding vasogenic oedema. The lesion was hyperintense to grey matter on the T2-weighted sequence. Histology at surgery was adenocarcinoma consistent with a breast primary

**Fig. 21:**
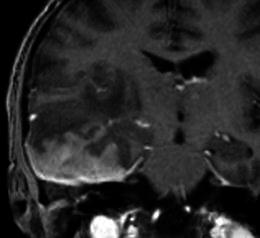
Post-contrast coronal T1-weighted sequence in a 63-year-old man with a remote history of follicular lymphoma demonstrates an extra-axial mass within the right middle cranial fossa with a broad dural attachment and dural thickening with intra axial extension into the right temporal lobe. There is homogeneous enhancement of the dural component and mildly heterogeneous enhancement of the intra-axial component. There is surrounding oedema and mass effect. Appearances could represent a meningioma with intra-axial extension, however were found to be lymphoma at histology

There are a number of neoplastic and non-neoplastic entities that may clinically and radiologically mimic meningiomas, notably haemangiopericytomas, metastases, lymphoma and neurosarcoid. Haemangiopericytomas are rare, WHO grade II neoplasms with a high local recurrence rate. In contrast to meningiomas, however, they do not calcify or result in hyperostosis of the adjacent calvarium but may cause direct skull erosion. They often demonstrate heterogeneous enhancement [18, 19], prominent internal flow voids and may have a narrow, stalk-like or broad dural attachment [18] (Fig. 19a and b). Dural metastases may mimic meningiomas and may have an enhancing dural tail; however, they are typically hyperintense in the T2-weighted sequence. They most frequently result from breast carcinoma (Fig. 20), adenocarcinomas, squamous cell carcinoma of the lung and renal cell carcinoma [19]. The presence of multiple lesions may aid diagnosis. Secondary CNS lymphoma may present as a dural-based lesion mimicking meningioma (Fig. 21) and spreads haematogenously. Lymphoma is typically isointense to hypointense in the T2-weighted sequence [18] and demonstrates intense post-contrast enhancement. Lesions may be multiple and also involve the leptomeninges. Sarcoidosis involves the CNS in 5 % of cases and may involve the dura but also the leptomeninges, perivascular subarachnoid spaces, cranial nerves and brain parenchyma. Lesions may be solitary and focal, mimicking meningiomas or diffusely infiltrating and are typically hypointense on T1-weighted sequences, hyperintense on T2-weighted sequences and demonstrate homogeneous post-contrast enhancement [19]. The coexistence of pulmonary sarcoid in the majority of cases aids diagnosis.

## Conclusion

Meningiomas are the commonest extra-axial tumour of the CNS and can have a varied appearance on imaging. There are a number of typical and atypical MRI features of meningiomas that are described and it is important for the reporting radiologist to have a broad understanding of their variable potential appearances in order to differentiate these lesions from the numerous lesions that can mimic their appearance.

## References

[1] Toh CH (2008). Differentiation between classic and atypical meningiomas with use of diffusion tensor imaging. AJNR Am J Neuroradiol.

[2] Whittle IR (2004). Meningiomas. Lancet.

[3] O’Leary S (2007). Atypical imaging appearances of intracranial meningiomas. Clin Radiol.

[4] Nagar VA (2008). Diffusion-weighted MR imaging: diagnosing atypical or malignant meningiomas and detecting tumor dedifferentiation. AJNR Am J Neuroradiol.

[5] Buetow MP, Buetow PC, Smirniotopoulos JG (1991). Typical, atypical, and misleading features in meningioma. Radiographics.

[6] Tokgoz N (2005). Primary intraosseous meningioma: CT and MRI appearance. AJNR Am J Neuroradiol.

[7] Paek SH (2005). Microcystic meningiomas: radiological characteristics of 16 cases. Acta Neurochir (Wien).

[8] Hakyemez B (2006). Meningiomas with conventional MRI findings resembling intraaxial tumors: can perfusion-weighted MRI be helpful in differentiation?. Neuroradiology.

[9] Elder JB (2007). Primary intraosseous meningioma. Neurosurg Focus.

[10] Komotar RJ, Keswani SC, Wityk RJ (2003). Meningioma presenting as stroke: report of two cases and estimation of incidence. J Neurol Neurosurg Psychiatry.

[11] Heye S (2006). Symptomatic stenosis of the cavernous portion of the internal carotid artery due to an irresectable medial sphenoid wing meningioma: treatment by endovascular stent placement. AJNR Am J Neuroradiol.

[12] Yue Q (2008). New observations concerning the interpretation of magnetic resonance spectroscopy of meningioma. Eur Radiol.

[13] Chen TY (2004). Magnetic resonance imaging and diffusion-weighted images of cystic meningioma: correlating with histopathology. Clin Imaging.

[14] Bitzer M (1998). Angiogenesis and brain oedema in intracranial meningiomas: influence of vascular endothelial growth factor. Acta Neurochir (Wien).

[15] Nakano T (2002). Meningiomas with brain edema: radiological characteristics on MRI and review of the literature. Clin Imaging.

[16] Bosnjak R (2005). Spontaneous intracranial meningioma bleeding: clinicopathological features and outcome. J Neurosurg.

[17] Filippi CG (2001). Appearance of meningiomas on diffusion-weighted images: correlating diffusion constants with histopathologic findings. AJNR Am J Neuroradiol.

[18] Chourmouzi D (2012). Dural lesions mimicking meningiomas: a pictorial essay. World J Radiol.

[19] Johnson MD (2002). Dural lesions mimicking meningiomas. Hum Pathol.

